# The Role of COVID-19 Vaccine Perception, Hope, and Fear on the Travel Bubble Program

**DOI:** 10.3390/ijerph19148714

**Published:** 2022-07-18

**Authors:** Eeman Almokdad, Kiattipoom Kiatkawsin, Mosab Kaseem

**Affiliations:** 1 Department of Hospitality and Tourism Management, Sejong University, Seoul 05006, Korea; eemanalmokdad@gmail.com; 2 Business Communication and Design Cluster, Singapore Institute of Technology, Singapore 138683, Singapore; 3 Department of Nanotechnology and Advanced Materials Engineering, Sejong University, Seoul 05006, Korea; mosabkaseem@sejong.ac.kr

**Keywords:** travel bubble, Vaccinated Travel Lane (VTL), COVID-19 pandemic, COVID-19 vaccine, hope, fear

## Abstract

The travel bubble program presented an appealing strategy for reopening international travel safely. However, a full vaccination regime is the foremost prerequisite of the program. Therefore, vaccination and the travel bubble are inextricably linked. This study investigated the roles of perceived vaccine efficacy, attitude towards the COVID-19 vaccine, and attitude toward the travel bubble on travel bubble intention. More importantly, the study also examined the mediating role of hope and fear among unvaccinated Korean adults between 20 and 29 years old. A total of 535 samples were collected to test the proposed conceptual model using structural equation modeling. In general, the results supported the proposed hypotheses. Notably, the intention to travel to a bubble destination was explained by 57% of the variance. Furthermore, hope mediated the relationship between vaccine attitude and travel bubble intention. Whereas fear mediated the relationship between perceived vaccine efficacy and intention. Hence, the findings suggest doubts around the vaccine efficacy and that a positive attitude towards the vaccine also install hope among the research samples.

## 1. Introduction

International tourism has been one of the worse affected industries by the COVID-19 pandemic. Reports estimated a decrease between 74 and 87 percent of international tourist arrivals compared to the pre-COVID periods globally [1,2]. Despite numerous efforts to revive tourism and its stakeholders by various governments such as promoting domestic tourism, providing subsidies, lowering taxes, and issuing travel vouchers, recovery has been limited [3,4,5]. The requirement to serve lengthy quarantines after arriving at some destinations was the most prominent barrier to most leisure tourists [1]. Consequently, governments and businesses have investigated the implementation of travel bubbles as a potential strategy to restart international tourism [6]. Travel bubbles is a general term referring to an agreement between two or more destinations to allow travel between them without the need to quarantine [7,8,9,10]. The program was open to all tourists who are fully vaccinated against COVID-19 [8,11,12].

For the travel bubble scheme to succeed, many residents must also be vaccinated even if they are not planning to travel to a bubble destination. The government’s aim should still be to reach herd immunity and have the local population be protected against any new surges in affected cases either locally or through the travel bubble initiative [13,14,15,16,17,18,19,20]. Hence, the travel bubble initiative may encourage more unvaccinated people to receive their vaccinations. Even though many regions have removed border travel restrictions and the vaccination rate has reached the target of 80% [14], the blueprint of such a program can still be beneficial to future events where individuals are required certain proof before their trips.

The present study identified the following antecedents that may help explain an individual’s intention to travel to a bubble destination. Individual behaviors often require a favorable attitude [21]. A positive attitude towards vaccination against COVID-19 is likely to also be affected by the belief that COVID-19 vaccines possess the efficacy to protect them against infection or severe symptoms [22]. Perceived vaccine efficacy or sometimes referred to as vaccine confidence was found as one of the main reasons individuals form a positive attitude towards the vaccine [1,22]. Then, a favorable attitude towards vaccination may also lead to a favorable attitude towards the travel bubble program as proof of vaccination is a critical component for partaking in the program. At the same time, the subjective evaluation or emotional responses to the overall pandemic situation can be immensely powerful in promoting engagement in various activities [23,24]. More specifically, due to the uncertain feelings developed by the pandemic’s ever-changing situations emotions such as hope (positive emotion) and fear (negative emotion), the travel bubble initiative could bring excitement to travelers and potentially restart international tourism [25,26,27]. In other words, the availability of vaccines and the travel bubble program may install a certain level of risk-taking (hope) in potential tourists or are they still risk-averse (fear) because of the pandemic [3,4,5].

Some of the recent research on tourism during COVID-19 focused on the travel bubble programs. However, they tend to focus on the general implementation of the program and the cooperation between all stakeholders [6,7,9]. On the other hand, studies that examined travel intention during the pandemic were mainly focused on perceived risks of infection. Thus, prompting researchers to examine mitigating behaviors such as compliance with social distancing guidelines [6,8,17,18]. Although some existing research may explain tourist intention to travel overseas during the pandemic, no attempt has been dedicated to investigating the specific antecedents of travel bubble intention comprehensively.

The present study aimed to fulfill the void in research on the travel bubble program by addressing the following three objectives. One is to identify relevant constructs that explain the influence of the COVID-19 vaccine and the travel intention to a bubble destination. Two is to enhance the explanatory power of the study model by investigating the roles of hope and fear. Three, to empirically test the proposed study model among unvaccinated Korean adults aged between 20 and 29 years old. At the time of the study, the vaccination rate in Korea was comparatively low among young adults and the travel bubble program was heavily under discussion by the media. The outcomes of this study would not only yield a deeper understanding of tourists’ intention toward traveling to bubble destinations but also establish clear and consistent communication to encourage individuals’ decisions to consider the travel bubble program. Ultimately, the travel bubble plan may provide a blueprint for a safe reopening of international travel as well as provide a vital strategy for any future crises.

## 2. Literature Review

### 2.1. Perceived Efficacy of COVID-19 Vaccine

Over the years, many viral illnesses have been effectively reduced by using vaccines. Past experiences found direct immunity of the population by vaccination is an effective way to reduce the rate of infectious cases, severe symptoms, and death [28]. However, the fast development of the COVID-19 vaccines, side effects, and mixed results from COVID-19 vaccines have created some doubts about the efficacy of the available vaccines [29]. Perceived vaccine efficacy is defined as the belief that a vaccine will reduce the likelihood of infection that can occur without the vaccine. It is the perceptual evaluation by individuals of vaccine effectiveness in preventing disease transmission [30].

Intentions to take any vaccines are fundamentally driven by the beliefs about the vaccines’ efficacy [31]. Several studies have found evidence that highly perceived vaccine efficacy eventually influences vaccine uptake [32]. For example, the intention to acquire influenza vaccines was primarily predicted by perceived vaccine efficacy [31,33,34,35]. At the same time, a study among Turkish health care workers found them reluctant to acquire vaccines due to the relatively minor protection from seasonal flu vaccines [36].

### 2.2. Attitude toward COVID-19 Vaccine

Attitude has been consistently used to describe the individuals’ favorable or unfavorable evaluation of an object [37,38]. It largely served as a basis for the explanation of the individual behavioral intention and is commonly used as a prediction of health behavior and health behavior intention [22,39]. In the context of this present study, attitude toward the COVID-19 vaccine has been defined as a cognitive evaluation that resulted in either a favorable or an unfavorable disposition toward the COVID-19 vaccine. Studies found barriers against the COVID-19 vaccine include fear of needles, concerns about side effects, concerns over vaccine development, and many more [1,40]. At the same time, perceived vaccine efficacy was widely reported as the most important factor in determining patient attitude [14,38].

Vaccine acceptance level (positive attitude) has often been studied from the risk-to-rewards perspective [22,33,41]. The reward is the protection an individual would develop after taking the vaccine, while the risks may include minor to severe side effects, and even death [35]. Central to this perspective is efficacy. The risk would be worth taking only if the reward is developing sufficient protection against a virus that is believed to be severe [40]. A study found that perceived vaccine efficacy was more effective in predicting vaccine uptake than perceived vaccine safety among U.S. residents [32]. Since the start of COVID-19 vaccination programs across many countries, the efficacy has often been a topic of debate. Comparisons of the efficacy level between different brands of vaccines have received much public attention [42]. Cases of reinfection even after receiving the full dosage of the vaccines have frequently been reported, further casting doubts over their efficacy [40]. In a study of influenza vaccine uptake among healthcare workers in Turkey, perceived low efficacy was the main reason for research samples forming a negative attitude towards vaccination [36]. When the booster vaccine program became mandatory due to the diminishing protection level [43]. Further, doubts about their efficacy were generated. Hence, it is critical to examine the effect of perceived vaccine efficacy concerning vaccine attitude in order to investigate further intentions that require proof of vaccination such as the travel bubble. Consequently, the following hypothesis was developed.

**H1.**  *Perceived efficacy of COVID-19 vaccine has a significant and positive impact on attitude towards COVID-19 vaccine.*

### 2.3. Hope

Hope is considered one of the positive psychological states that reflects inner pleasant emotions [27]. Hope also has a motivating capacity and integrates into individuals’ goals and their strategies to achieve those goals [44]. In the seminal work by Snyder [45], the hope theory feathers two components. The first component is the agency which explains one’s ability to achieve desired goals. The second component is pathways. They represent the plans or strategies one believes may lead to the desired goals. During the prolonged COVID-19 pandemic, people tend to form a pool of negative emotions such as fear of infection and resentment [1]. However, the availability of COVID-19 vaccines and the travel bubble initiative may have offered hope that life may return to normal and international travel could resume.

In positive psychological theories, hope and efficacy were found to be closely related [46]. When individuals’ perception of efficacy is high, they create more agency and pathways to deal with stressful situations [47]. The various social distancing measures may have created stressful and undesirable situations but acquiring vaccines and traveling to a bubble destination may present a goal an individual may look forward to participating As a result, confidence in the vaccine efficacy is fundamental in creating a hopeful mindset amidst the pandemic. Empirical studies have also supported the role of efficacy as an antecedent of hope. A study of adolescents suffering from cancer found their hope level increased when they believe in the efficacy of their treatment [48]. Another study in Poland found doctors have increased optimism and hopefulness in relation to the pandemic after receiving their COVID-19 vaccines [26].

Similar to the relationships between efficacy and hope, a positive attitude was also found to have a strong influence over hopeful feelings [27]. A study by Lukoff [49] found a positive attitude to life among cancer patients has led them to have more hope, and that significantly assists them to cope with their illness. At the same time, having a hopeful attitude can prevent psychological issues such as depression [50]. Research found that the introduction of the COVID-19 vaccines created a positive attitude in overcoming the pandemic among both nurses and patients [27]. The importance of family and community created a strong desire to have closer interactions with them after a prolonged period of social distancing and isolation [22]. Consequently, the availability of the vaccine and the travel bubble programs may install a hopeful attitude toward the COVID-19 pandemic. The following hypotheses were developed to reflect the discussed relationships between vaccine efficacy, attitude, and hope.

**H2.**  *Perceived efficacy of the COVID-19 vaccine has a significant and positive impact on hope.*

**H3.**  *Attitude towards the COVID-19 vaccine has a significant and positive impact on hope.*

### 2.4. Fear

Contrary to hope, fear reflects negative emotions and represents an awareness of danger [51]. Fear is described as a natural response to a present threat. It is a defensive reaction that often motivates individuals to take evasive actions in the face of danger [52]. Fear is an emotional construct that emerges when individuals assess risk and it is a critical determinant of individual behaviors [5]. In the present study, fear of the COVID-19 situation refers to the holistic threat perception of the pandemic situation.

The consequence of the pandemic on daily lives includes social distancing measures preventing large social gatherings and has eliminated many leisure activities such as international tourism [19]. Although solutions such as vaccines, better treatments, and improved hygienic practices were introduced, the overall situation has not necessarily improved [1]. The multiple waves of infected cases emerged due to new variants of the virus caused reimposing of strict social distancing measures that have created a certain level of pessimism among the public [6]. Consequently, individuals may develop a high level of fear and pessimism in general daily situations, and they may be more reluctant to engage in activities they deem could worsen the situation such as traveling.

Previous studies highlighted the significance of perceived efficacy in alleviating fearful emotion [39]. Perceived efficacy is essential in individuals’ appraisal of coping mechanisms [53]. In other words, individuals may feel less fearful of the COVID-19 pandemic if they believe that the available vaccines have the ability to prevent infections or reduce the symptom severity [23]. Although many were optimistic about the vaccines, their remarkable pace of development and mixed early results have installed some vaccine safety concerns [40,54]. In addition, the perceived risk of side effects was often cited by those who were hesitant to acquire vaccinated [55]. Thus, the study posits the influence of perceived vaccine efficacy on fear.

Previous studies [25,56] have further demonstrated the relationship between attitude and fear. Attitude towards the COVID-19 vaccine was thought to be a result of risk versus benefits [25]. Hence, attitude toward vaccination could affect individuals’ desire to be vaccinated and may develop or maintain a certain level of fear towards the pandemic situation. Ultimately, fear may outweigh the desire to engage in non-essential activities such as travel. Thus, fearful individuals would be less inclined to participate in the travel bubble program. Two further hypotheses were developed to reflect the discussion in this section.

**H4.**  *Perceived efficacy of COVID-19 vaccine has a significant and negative impact on fear.*

**H5.**  *Attitude toward the COVID-19 vaccine has a significant and negative impact on fear.*

### 2.5. Attitude toward Travel Bubble

The travel bubble program is a state-level agreement that enables quarantine-free international air travel between at least two countries based on a mutually agreed set of public health mitigation measures [3,12]. The initial framework was introduced before the availability of vaccines and had a strict operating guideline [7,8]. For example, one of the first travel bubble frameworks was proposed by the Taiwanese government. It required tourists to travel only in the same groups and all activities including meals, shopping, and sightseeing must be planned and approved in advance and must be carried out without the presence of local residents. Transportation and hotels must be designated by the tour agencies and receive government approvals before arrival [7]. These restrictions removed any interaction between the tourists and the local environment, thus, eliminating the key appeal of international travel. As a result, this framework never came to fruition.

The availability of the COVID-19 vaccine was soon followed by a revision of the travel bubble concept where vaccination plays a central role [1]. Subsequently, the issuance of vaccine passes, or proof of vaccination by many countries have allowed a certain level of normal daily life to return [9,57]. Therefore, the revised travel bubble program should make traveling to another country during COVID-19 more feasible for a wider range of people in the market [10]. In July 2021, the Korean government introduced its version of the travel bubbles framework. It was officially referred to as the safe travel zone framework with nations that had largely contained the spread of infection. Korea began its travel bubble program with only a select group of destinations, including Singapore, Thailand, Taiwan, Guam, and Saipan. Later the program expanded to even more destinations [13]. Two main prerequisites for tourists were, one, they must be fully vaccinated against COVID-19, and two, they are required to produce negative test results for COVID-19 before departure and upon arrival. In turn, tourists are free to participate in all activities open to the local population [1,6].

The attitude towards the travel bubble represents a result of the evaluation of whether the individuals feel favorable or unfavorable towards the travel bubble program. Although the travel bubble program makes short-term international travel more feasible, it does not necessarily mean people will perceive it positively. There are still concerns over how the travel bubble may import cases from abroad or receive treatment abroad in case of being infected while traveling [1,7,8]. A full vaccination regime is still the prerequisite to participating in the travel bubble program [10]. Additionally, both the vaccination program and the travel bubble program share the same goal in that they aid the return of social interactive activities. Hence, the study posits individuals who have a positive attitude toward vaccines may also have a positive attitude toward the travel bubble program as reflected in the following hypothesis.

**H6.**  *Attitude towards COVID-19 vaccine has a significant and positive impact on attitude towards travel bubble.*

### 2.6. Travel Bubble Intention

The intention is the individual’s propensity to perform a given behavior [58]. The intention construct is a mental state that indicates the readiness of individuals to perform future behaviors and it has been widely used as a key predictor of future behaviors [59]. In this study, the travel bubble intention is the individuals’ propensity to participate in the travel bubble program. Attitude has been a reliable proxy of behavior intention in many tourism-related contexts [8,60]. Therefore, individuals’ intention to travel to a bubble destination would likely be a consequence of a favorable attitude towards the program. Additionally, the travelers must also fulfill two essential requirements, one, they must be fully vaccinated against COVID-19, and two, they can produce negative test results before departure and upon arrival.

**Figure 1:**
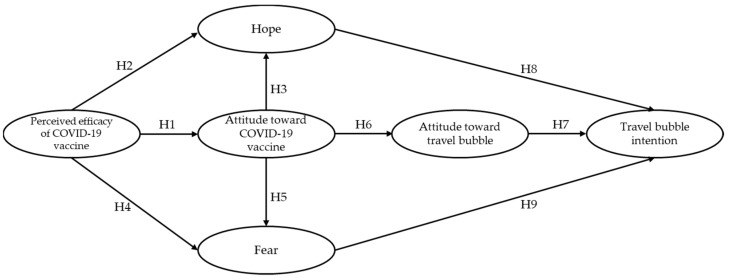
Proposed conceptual model.

Any traveling activities during the COVID-19 pandemic are likely to be affected by a certain level of subjective evaluation [18]. Fear had especially been proven as a critical factor hindering people’s willingness to take a trip [61]. At the same time, the advent of vaccines, travel bubbles, and other measures aimed to bring normality back to daily lives may have brought a level of hope back to individuals [19]. Past research suggested that consumer decisions are often driven by a combination of cognitive reasoning as well as subjective evaluation [8,38]. Past research found that cognitive constructs such as attitude and behavioral intention can be mediated by subjective evaluation such as emotions [38]. The addition of emotional constructs as mediators between attitude and intention was found to enhance the explanatory power of intention [38,60]. Hence, the subjective evaluation constructs, hope, and fear are hypothesized to mediate the relationships between the perception of vaccines and travel bubble intention. The final three hypotheses were developed as follows and the relationships among study variables were illustrated in Figure 1.

**H7.**  *Attitude towards the travel bubble has a significant and positive impact on travel bubble intention.*

**H8.**  *Hope has a significant and positive impact on travel bubble intention.*

**H9.**  *Fear has a significant and negative impact on travel bubble intention.*

## 3. Methods

### 3.1. Measurement Items

All measurement items have been adopted from previous studies. The items were then rephrased to reflect the context of the study. A total of six latent constructs were measured using a total of 24 items. Whereas the measurement items used to measure the perceived efficacy of the COVID-19 vaccine were adopted from Wang et al. [62]. The attitude towards the COVID-19 vaccine construct was measured using measurement items adopted from Kiatkawsin et al. [38]. The items used to measure hope as well as fear were adopted from Kim et al. [63]. Lastly, attitude towards travel bubbles and travel bubbles intention were measured using items from Lou and Lam [8]. The list of all measurement items can be seen in Appendix A. The items were measured using 7-point Likert-type scales ranging from (1) strongly disagree to (7) strongly agree.

### 3.2. Survey Development

A cover letter was included to provide a brief description of the study context and a short explanation of the travel bubbles program was provided. Two screening questions were added to assure the survey participants were appropriate for the research objectives. One, they must not be vaccinated against COVID-19, and two, are aged between 20 and 29 years old at the time of completing the survey. In the last part of the survey, demographic questions were added. The final version of the survey was subjected to pre-testing among senior academics and several people in the same age group for general comprehension of the survey. Only minor comments were received, and adjustments were made before finalization. The English version of the survey was then translated into Korean by a native bilingual speaker of both languages. Another round of pretesting was conducted among Korean speakers to ensure general comprehension of the Korean version as well as translation accuracy.

### 3.3. Sampling and Data Collection

A convenience sampling technique was adopted. The target samples were the general Korean public aged between 20 and 29 years old who had not taken the COVID-19 vaccine at the time of the data collection period on the first week of August 2021. At the time of the study, unconfirmed news about the safety of the COVID 19 vaccine has aroused hesitancy among the general public in South Korea. Specifically, reports have revealed that a considerable portion of young Koreans have preferred to continue social distancing measures rather than receive the vaccine because they are unsure about its safety [42]. Moreover, at that time, the vaccination program in Korea was not extended to the general public under 30 years old. Hence, they were in a suitable situation to demonstrate their perception of the COVID-19 vaccines as well as the travel bubble program which heavily relied on the vaccination rate [16,64].

The survey was distributed to a pool of panel samples by Embrain, a reputable research agency based in Korea. The company is a leader in providing only research solutions and was the agency of choice in other empirical research projects. A total of 545 completed surveys were collected. They were screened for missing data, unengaged responses, normality, and outliers. No cases with missing data were but 10 cases with evidence of unengagement were removed. The remaining 535 cases provided skewness scores ranging between −0.847 and 0.694. The kurtosis scores were between −0.713 and 0.795. Hence, no violation of data normality and outliers were identified. A summary of the sample profiles can be seen in Table 1.

## 4. Results

### 4.1. Confirmatory Factor Analysis

The structural equation modeling analysis followed Anderson and Gerbing’s [65] two-step approach. Firstly, confirmatory factor analysis (CFA) had been performed to examine the fit of data with the measurement model. The results yielded satisfactory goodness-of-fit statistics result (x^2^ = 1046.666, *df* = 325, *p* < 0.01, x^2^/*df* = 3.221, RMSEA = 0.064, CFI = 0.957, IFI = 0.957). The data was also reliable with the composite reliability (CR) scores ranging from 0.889 to 0.966 which satisfied the minimum requirement of 0.7 [66]. The convergent validity was also established in all constructs with the average variance extracted (AVE) scores all higher than 0.5 [67]. AVE scores were between 0.732 and 0.848. Lastly, the correlation scores were lower than the square root of the AVE indicating the discriminant validity of the measurement model has been established. The result of CFA has been summarized in Table 2.

### 4.2. Structural Equation Modeling

After validation of the measurement model, an examination of the structural model also yielded satisfactory goodness-of-fit statistics (x^2^ = 817.526, *df* = 239, x^2^/*df* = 3.421, RMSEA = 0.067, CFI = 0.959, IFI = 0.959, TLI = 0.953, NFI = 0.944, PCFI = 0.831). Next, path analysis was conducted to test the proposed hypotheses. All but two of the proposed hypotheses were supported as summarized in Table 3. The influence of perceived efficacy on hope was not significant. In addition, the effect of attitude toward the COVID-19 vaccine to fear did not yield a significant result. Furthermore, 67.8% of the variance explained the attitude towards the COVID-19 vaccine construct, the most among the study variables. The final construct, travel bubble intention, was explained by 57% of the variance. Attitude towards travel bubble produced the largest amount of impact on travel bubble intention while attitude towards COVID-19 vaccine produced the second-largest total impact on intention.

**Figure 2:**
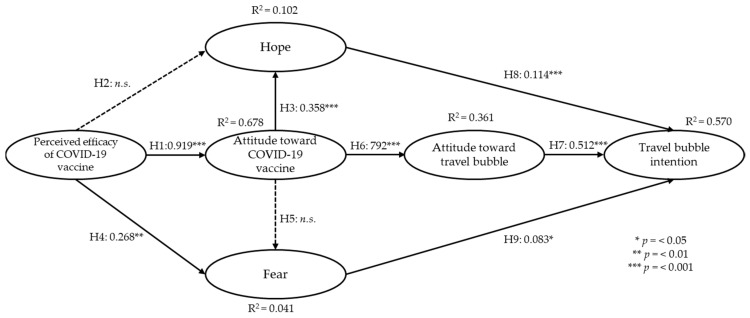
Conceptual model and the results of structural equation modeling.

The indirect effect assessment was conducted to further examine the type of mediation within the proposed model. The bootstrapping method was used with 2000 resampling amounts and at 95 percentile confidence intervals. The results found that attitude toward COVID-19 vaccine was a complete mediator between the perceived efficacy of COVID-19 vaccine and hope. The other indirect paths were significant. Thus, indicating a partial mediating role among the variables. The summary of the indirect effect assessment results is presented in Table 4. In addition, the conceptual model with SEM results has been included in Figure 2.

## 5. Discussion

### 5.1. General Discussion

In general, the research findings have reaffirmed previous studies’ results. Perceived vaccine efficacy contributed strongly to the formation of vaccine attitude in the case of COVID-19, which was consistent with studies of other vaccines in the past [31,32,33,34,35]. Likewise, the results showed that a favorable attitude towards the COVID-19 vaccine led to a favorable attitude towards the travel bubble program. Eventually, attitude toward a travel bubble help predicts the intention to travel to a bubble destination. The effect of attitude on intention is consistent with many previous studies [8,60]. Importantly, the results suggested that perceived vaccine effectiveness is a critical element in the strategy to reopen international tourism using the travel bubble program. On the other hand, the travel bubble could be used to stimulate the appeal of vaccine uptake among the young population.

The results of the current study highlighted that hope and fear are major constructs in the coping strategy associated with uncertain situations, such as traveling during the COVID-19 pandemic. The results could be interpreted that the COVID-19 vaccine has installed a certain level of hope among the study samples. Soon after the data collection period, the vaccine was made available to all adults over the age of 18. The vaccine uptake among 18–29 years old was slow upon availability, but adjustments to social distancing measures have helped increase vaccine uptake in this age group substantially. The adjustments included relaxation of restrictions when dining out at restaurants and bars, and visiting leisure establishments such as hotels, cinemas, and religious gatherings. Importantly, the relaxation was only applicable to those who have been vaccinated. Given that international travel is a form of serious leisure activity, the announcement of the travel bubble initiative can be compared favorably to the relaxation of social distancing measures. The results supported this assumption in that vaccine efficacy and attitude increased the level of hope and it showed a strong indication to support the travel bubble program.

While fear mediates the relationship between efficacy and intention, both relationships were positive rather than negative as hypothesized. Hence, instead of vaccine efficacy reducing the amount of fear as suggested by some studies [55,68], the finding suggested that as efficacy increases, fear also increases. Then, as fear increases, the travel bubble intention also increases. The unexpected outcomes could be attributed to two factors. The first factor could be that negative anticipated emotions such as fear do play a major role in travel intentions [69]. In other words, when people plan their travels, they mostly anticipate positive experiences although they are aware that negative experiences may arise. The second factor is due to how tourists may still feel fearful of the pandemic but the anticipation of joyful experiences that may be derived from traveling outweighs the fear [70,71]. In this context, feeling fearful while still showing intention to travel suggests a precautious mindset on the travel bubble program.

### 5.2. Implications

One of the key contributions of this study is how the travel bubble should be a viable strategy for a safe reopening of international travel from the demand side. It can also encourage people reluctant to acquire vaccinated to opt into the program. Although Korea and other destinations have abolished most border controls rendering the travel bubble program redundant. It is still important to acknowledge that the travel bubble program can be a strategy to implement in case of a future pandemic breakout where cross-border travel is only permitted based on certain conditions. The technologies and experience gained from this pandemic would allow a prompter implementation to prevent a severe impact on tourism businesses. Thus, the blueprint of the travel bubble will be especially helpful for destinations that traditionally have been relying on international tourism arrivals as their main source of livelihood.

The findings on fear have also sparked a vital view of the travel bubble program in that even after the easing of restrictions and restarting of international tourism; it is still possible that tourists may contract COVID-19 at some point in their travel. Moreover, since the outbreak of the COVID-19 pandemic, tourists’ psychological attitudes have dramatically changed, and the travel pattern has become more selective. Individuals became more cautious about the risk associated with travel. Therefore, a travel bubble program can be a viable paradigm during and post pandemic-restricted travel.

Moreover, the travel bubble program framework could be applicable in the countries that have ever suffered from infectious diseases to increase their tourism demand. In other words, the travel bubble program could be a pivotal strategy for international travel in order to protect travelers visiting endemic areas as well as to prevent the importation of the disease to non-endemic areas. In addition, digging into the past, serious diseases such as COVID-19 have repeatedly occurred due to environmental changes and it is possible to be repeated in the future. Therefore, the travel bubble program could be a practical blueprint for the fast recovery of the travel industry during disease outbreaks in the future.

In addition, the study contributed theoretically to reaffirming the relationships between the perception of COVID-19 vaccine efficacy, attitude toward the vaccine, attitude towards the travel bubble, and eventually, intention. More importantly, the study provided significant insights into the effects of positive and negative emotions namely fear and hope, in the time of a global health crisis. The conceptual framework of this study provides a basis for understanding tourist behaviors when presented with a new strategy to resolve international travel restrictions.

### 5.3. Limitations and Recommendations for Future Research

This study is not free from limitations. The first limitation is that the data used in this study only represented a small group of samples. In addition, the findings may not be applicable to other countries and age groups as the data specifically targeted unvaccinated Korean adults. Hence, the study’s lack of generalizability should present an avenue for future research to further explore the tourist perception of the travel bubble program in other contexts. Future research projects looking to validate the conceptual model are highly encouraged. The study also feels that the travel bubble program consists of many facets and stakeholders. Therefore, future projects may look into further examining related variables such as the role of governments, service providers, and tour operators at the time of a pandemic.

## 6. Conclusions

The present study aimed to investigate the antecedents of intention to travel to travel bubble destinations. The travel bubble program was developed as a strategy to allow quarantine-free travel between two or more international destinations. Travelers in the bubble programs must fulfill two essential requirements. They need to be fully vaccinated and able to produce negative test results before departing the origin country and upon arrival in the destination country. Due to the requirement to be fully vaccinated, it is then become critical to understand the role of vaccine perception and its influence on the travel bubble intention. The study identified the following constructs as antecedents, perceived COVID-19 vaccine efficacy, attitude toward COVID-19 vaccine, attitude toward travel bubble, and two affective responses (hope and fear). Research samples include 535 Korean adults aged between 20–29 who did not take any COVID-19 vaccines at the time of their participation in the research were used to test the proposed study model. The findings suggested that vaccine efficacy was a crucial determinant of a positive attitude toward the COVID-19 vaccine and subsequent constructs. Overall, the travel bubble intention was explained by 57% of the variance. Thus, the study provided empirical evidence that a travel bubble framework can be an effective strategy to help cross-border movement of people even when there are critical conditions imposed. In future crises, destinations may wish to deploy this framework early to minimize travel disruptions given the experience and available technologies. Specifically, destinations can leverage technologies such as contact tracing, digital identification, status monitoring, and more to promptly execute the bubble strategy. Tourism-related businesses may take the opportunity to integrate some of the technologies to enhance their operations. For example, tour agencies and cruise companies can use applications to track their tour members during their trips. It can provide a convenient communication channel as well as help avoid leaving tour members behind. In this light, future research is encouraged to investigate how some of the technologies being implemented in the bubble framework can be utilized post-pandemic.

## Figures and Tables

**Table 1:** Demographic information.

Variable	Category	Distribution	Valid Percentage
Gender	Male	255	47.7
	Female	280	52.3
Age	Mean	25.57	
Area of residential	Seoul	143	26.7
	Gyeonggi-do	149	27.9
	Busan	40	7.5
	others	203	37.9
Marital Status	Single	510	95.3
	Married with children	8	1.5
	Married with children	17	3.2
Educational Background	High school or below	154	28.8
	Bachelor’s degree	334	62.4
	Master’s degree	15	2.8
	Doctorate	1	0.2
	Others	31	5.8
Usual traveling per year	1 time	415	77.6
	2 times	80	15.0
	3 times	24	4.5
	More than 4 times	16	3.0
Reduced travel trips during COVID 19 pandemic	Yes	438	81.9
	No	97	18.1
Living companions at home	Alone	110	20.6
	1 other person	76	14.2
	2 other people	121	22.6
	3 other people	184	34.4
	4 other people	44	8.2

**Table 2:** Summary of the confirmatory factor analysis results.

	AV	HP	FR	PE	ATB	TBI
**AV**	**0.909 ^b^**					
**HP**	0.320 ^a^	**0.882**				
**FR**	0.130	0.132	**0.856**			
**PE**	0.815	0.244	0.196	**0.882**		
**ATB**	0.589	0.177	0.124	0.554	**0.921**	
**TBI**	0.626	0.229	0.150	0.451	0.741	**0.916**
**AVE**	0.825	0.779	0.732	0.778	0.848	0.839
**CR**	0.966	0.913	0.889	0.955	0.944	0.940

Note 1. Goodness-of-fit statistics: x^2^ = 1046.666, *df* = 325, *p* < 0.01, x^2^/*df* = 3.221, RMSEA = 0.064, CFI = 0.957, IFI = 0.957. Note 2. AV = Attitude towards COVID-19 vaccine, HP = Hope, FR = Fear, PE = Perceived efficacy of the COVID-19 vaccine, ATB = Attitude towards travel bubble, TBI = Travel bubble intention. ^a^ Correlations; ^b^ Squared root of AVE are along the diagonal and in bold.

**Table 3:** Summary of the structural equation modeling results.

			Standardized Estimate	*t*-Value
**H1:** Perceived efficacy of COVID-19 vaccine	**→**	Attitude towards COVID-19 vaccine	0.919	24,178 ***
**H2:** Perceived efficacy of COVID-19 vaccine	**→**	Hope	−0.083	−0.931
**H3:** Attitude toward COVID-19 vaccine	**→**	Hope	0.358	4.499 ***
**H4:** Perceived efficacy of COVID-19 vaccine	**→**	Fear	0.269	3.244 **
**H5:** Attitude toward COVID-19 vaccine	**→**	Fear	−0.081	−1.110
**H6:** Attitude toward COVID-19 vaccine	**→**	Attitude toward travel bubble	0.792	18.953 ***
**H7:** Attitude toward travel bubble	**→**	Travel bubble intention	0.512	15.090 ***
**H8:** Hope	**→**	Travel bubble intention	0.114	3.393 ***
**H9:** Fear	**→**	Travel bubble intention	0.083	2.490 *
Goodness-of-fit statistics (Final model): x^2^ = 817.526 *df* = 239, x^2^/*df* = 3.421, RMSEA = 0.067, CFI = 0.959, IFI = 0.959, TLI = 0.953, NFI = 0.944, PCFI = 0.831, * *p* < 0.001	Total variance explained:		Total impact on travel bubble intention	
	R2 of AV = 0.678		PE = 0.396	
	R2 of HP= 0.102		AV = 0.466	
	R2 of FR = 0.041		ATB = 0.715	
	R2 of ATB = 0.361		FR = 0.078	
	R2 of TBI = 0.570		HP = 0.114	

Note. AV = Attitude towards COVID-19 vaccine, HP = Hope, FR = Fear, PE = Perceived efficacy of the COVID-19 vaccine, ATB = Attitude towards travel bubble, TBI = Travel bubble intention. * *p* ≤ 0.05, ** *p* ≤ 0.01, *** *p* ≤ 0.001.

**Table 4:** Summary of indirect effect assessment results.

Indirect Effect of	On			
	HP	FR	ATB	TBI
PE	0.313 **	−0.076	0.495 **	0.396 **
AV	-	-	-	0.466 **

Note. AV = Attitude towards COVID-19 vaccine, HP = Hope, FR = Fear, PE = Perceived efficacy of the COVID-19 vaccine, ATB = Attitude towards travel bubble, TBI = Travel bubble intention. * *p* ≤ 0.05, ** *p* ≤ 0.01, *** *p* ≤ 0.001.

## Appendix A

**Table A1:** Measurement Items.

Constructs	Measurement Items
Vaccine efficacy	Vaccination is a very effective way to protect me against COVID-19. It is important that I get the COVID-19 vaccine. Vaccination greatly reduces my risk of contracting COVID-19. The COVID-19 vaccine plays an important role in protecting my life and that of others. The contribution of the COVID-19 vaccine to my health and well-being is very important. Getting the COVID-19 vaccine has a positive influence on my health.
Vaccine attitude	I think taking the COVID-19 vaccine is a sensible choice. Following the Korean government’s recommendation to take the COVID-19 vaccine would protect me from getting seriously ill. I think that following the Korean government’s recommended COVID-19 vaccination program is the correct choice. Following the Korean government’s recommendation to take the COVID-19 vaccine will make me feel safe. I believe that following the Korean government’s recommended COVID-19 vaccination program is an intelligent choice. Following the Korean government’s recommendation to take the COVID-19 vaccine will make me feel calm.
Travel bubbles attitude	A travel bubble is an effective way to resume international travel. The Travel bubble concept can play an important role in resuming international travel. Organizing travel bubbles has a positive influence on a safe opening of international travel.
Travel bubbles intention	After vaccination against COVID-19, I would like to travel to the travel bubble destinations sometime in the future. I prefer to travel to a travel bubble destination compared to other destinations after vaccination against COVID-19 I will recommend the travel bubble destination to friends and family after they get the COVID-19 vaccine.
Hope	Now, when thinking about the COVID-19 situation, I feel hopeful. Now, when thinking about the COVID-19 situation, I feel optimistic. Now, when thinking about the COVID-19 situation, I feel encouraged.
Fear	Now, when thinking about the COVID-19 situation, I feel fearful. Now, when thinking about the COVID-19 situation, I feel afraid. Now, when thinking about the COVID-19 situation, I feel scared.
